# Identification of CD14 as a potential biomarker of hepatocellular carcinoma using iTRAQ quantitative proteomics

**DOI:** 10.18632/oncotarget.18782

**Published:** 2017-06-28

**Authors:** Jiao Guo, Rui Jing, Jian-Hong Zhong, Xin Dong, Yun-Xi Li, Yin-Kun Liu, Tian-Ren Huang, Chun-Yan Zhang

**Affiliations:** ^1^ Experimental Department, Affiliated Tumor Hospital of Guangxi Medical University, Nanning, Guangxi, PR China; ^2^ Hematology Department, Affiliated Hospital of Binzhou Medical University, Yantai, Shandong, PR China; ^3^ Hepatobiliary Surgery Department, Affiliated Tumor Hospital of Guangxi Medical University, Nanning, Guangxi, PR China; ^4^ Oncology Department, Affiliated Hospital of Binzhou Medical University, Yantai, Shandong, PR China; ^5^ Cancer Registry Department, People’s Hospital of Fusui County, Fusui, Guangxi, PR China; ^6^ Liver Cancer Institute, Zhongshan Hospital, Fudan University, Yangpu, Shanghai, PR China

**Keywords:** hepatocellular carcinoma (HCC), diagnosis, biomarker, iTRAQ, CD14

## Abstract

Hepatocellular carcinoma (HCC) is one of the most common malignant tumors without effective diagnostic biomarkers. This study intended to dynamically analyze serum proteomics in different pathological stages of liver diseases, and discover potential diagnostic biomarkers for early HCC. Patients with hepatitis B virus (HBV) infection, liver cirrhosis (LC), or HCC together with healthy controls (HC) were enrolled. Proteins differentially expressed between groups were screened using isobaric tagging for relative and absolute quantitation (iTRAQ), and promising HCC biomarker candidates were subjected to bioinformatics analysis, including K-means clustering, gene ontology (GO) and string network analysis. Potential biomarkers were validated by Western blotting and enzyme-linked immunosorbent assay (ELISA), and their diagnostic performance was evaluated using receiver operating characteristic (ROC) curve analysis. Finally, 93 differentially expressed proteins were identified, of which 43 differed between HBV and HC, 70 between LC and HC, and 51 between HCC and HC. Expression levels of gelsolin (GELS) and sulfhydryl oxidase 1 (QSOX1) varied with disease state as follows: HC < HBV < LC < HCC. The reverse trend was observed with CD14. These iTRAQ results were confirmed by Western blotting and ELISA. Logistic regression and ROC curve analysis identified the optimal cut-off for alpha-fetoprotein (AFP), CD14 and AFP/CD14 was 191.4 ng/mL (AUC 0.646, 95%CI 0.467-0.825, sensitivity 31.6%, specificity 94.4%), 3.16 ng/mL (AUC 0.760, 95%CI 0.604-0.917, sensitivity 94.7%, specificity 50%) and 0.197 ng/mL (AUC 0.889, 95%CI 0.785-0.993, sensitivity 84.2%, specificity 83.3%) respectively. In conclusion, Assaying CD14 levels may complement AFP measurement for early detection of HCC.

## INTRODUCTION

Hepatocellular carcinoma (HCC) is one of the most common malignant tumors, ranking sixth in morbidity and third in mortality worldwide [1, 2]. The high frequency of early metastasis means that HCC is usually at an advanced stage when diagnosed, reducing the likelihood that the patient can benefit from curative surgical treatment. Therefore, early detection of HCC, such as through assay of serum biomarkers, is of tremendous importance.

Serum alpha-fetoprotein (AFP), although the most widely used biomarker for HCC diagnosis in the clinic, is less than satisfactory because of its low specificity and sensitivity [3]. Numerous other candidate HCC biomarkers have been proposed, including lens culinaris agglutinin reactive AFP (AFP-L3), des-γ-carboxy prothrombin (DCP), prothrombin produced by vitamin K absence or antagonism II (PIVKA II), Golgi protein-73 (GP73), glypican-3 (GPC3), γ-glutamyl transferase (GGT), α-L-fucosidase (AFU), and alpha1-antitrypsin and apolipoprotein J (Apo-J) [4-8]. All have substantial shortcomings that severely limit their clinical usefulness. Studies are needed to identify novel HCC biomarkers with superior early diagnostic potential.

Isobaric tagging for relative and absolute quantitation (iTRAQ) has emerged as a powerful proteomic technology for sensitively and accurately searching for tumor biomarkers [9]. This technique relies on identifying proteins differentially expressed between, for example, a healthy and disease state, thereby allowing identification of disease markers. The traditional method of identifying differentially expressed proteins is two-dimensional gel electrophoresis, but this is poorly suited for detecting proteins that have a high molecular weight, are highly acidic or basic, or reside in the cell membrane [10]. The iTRAQ technique does not have such limitations, and it offers high throughput: proteins from up to 8 samples can be analyzed and quantified simultaneously [9, 11]. In addition to being simple and sensitive, the technique shows good repeatability, with a coefficient of variation between 0.04 and 0.14 [12]. When coupled with liquid chromatography-tandem mass spectrometry (LC-MS/MS), iTRAQ has proven successful at identifying disease-specific biomarkers and targets [13]. Here we used the iTRAQ technique coupled with LC-MS/MS to identify candidate serum biomarkers for early detection of HCC.

Guangxi is a high endemic area of HCC in China, and further we have established a high-risk population cohort comprised of more than 3,800 members with nested case-control study method since 2003. Serum samples from these individuals have been regularly collected since 2003, together with their medical status, including the results of early cancer screening and detection [14]. This specimen bank therefore provides the possibility of identifying serum biomarkers closely linked with HCC, as well as assessing their potential involvement in HCC onset and progression, especially the transition from HBV infection to HCC. We therefore applied the iTRAQ technique coupled with LC-MS/MS to identify candidate serum biomarkers for early detection of HCC based on the serum samples of high-risk population cohort.

## RESULTS

### Study population

**Figure 1:**
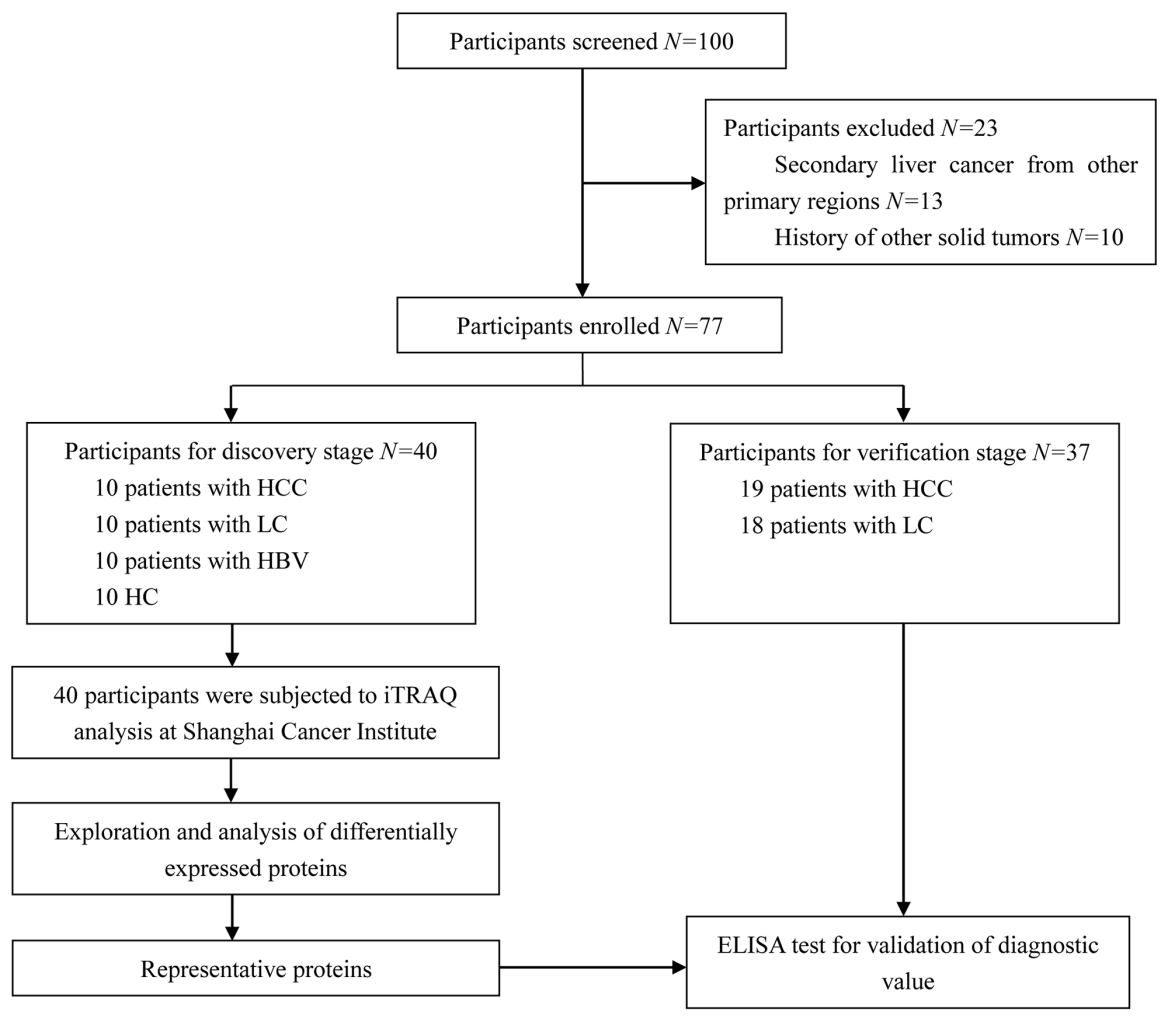
Flow diagram of participant screening and inclusion HC: healthy controls; HBV: hepatitis B virus infection; LC: liver cirrhosis; HCC: hepatocellular carcinoma.

A total of 100 participants were screened initially, and 23 participants were excluded because they had secondary liver cancer that had metastasized from other primary regions, or they had a history of other solid tumors. In the end, 77 participants were enrolled, of whom 40 participated in the discovery phase (10 each with HCC, LC, HBV, or HC). The remaining 37 participated in the validation phase: 19 with HCC and 18 with LC (Figure 1). The cause of LC patients was HBV infection and the cause of HCC patients was liver cirrhosis developed from HBV. The detailed demographic and clinical characteristics of participants were shown in Table 1.

**Table 1:** Demographic and clinical characteristics of participants

		HCC	LC	HBV	HC
Screening stage	Gender (Male : Female)	8:2	7:3	6:4	7:3
	Age (years)	50 (35-61)	45.5 (31-63)	41.1 (36-47)	50.1 (37-60)
	HBV DNA (IU/mL, log)	4.27±1.01	4.12±1.2	3.4±0.93	/*
	Treatment Status	N	N	N	/
Validation stage	Gender (Male : Female)	14:5	11:7	/	/
	Age (years)	50.3 (37-59)	47.6 (34-56)	/	/
	HBV DNA	4.3±1.04	4.6±1.01	/	/
	Treatment Status	N	N	/	/

HCC, hepatocellular carcinoma; LC, liver cirrhosis; HBV, hepatitis B virus infection; HC, healthy controls. N, receive no treatments; /, not applicable; *, HBV DNA levels of healthy controls were all lower than the minimum limit of detection.

### Depletion of high-abundance serum proteins

**Figure 2:**
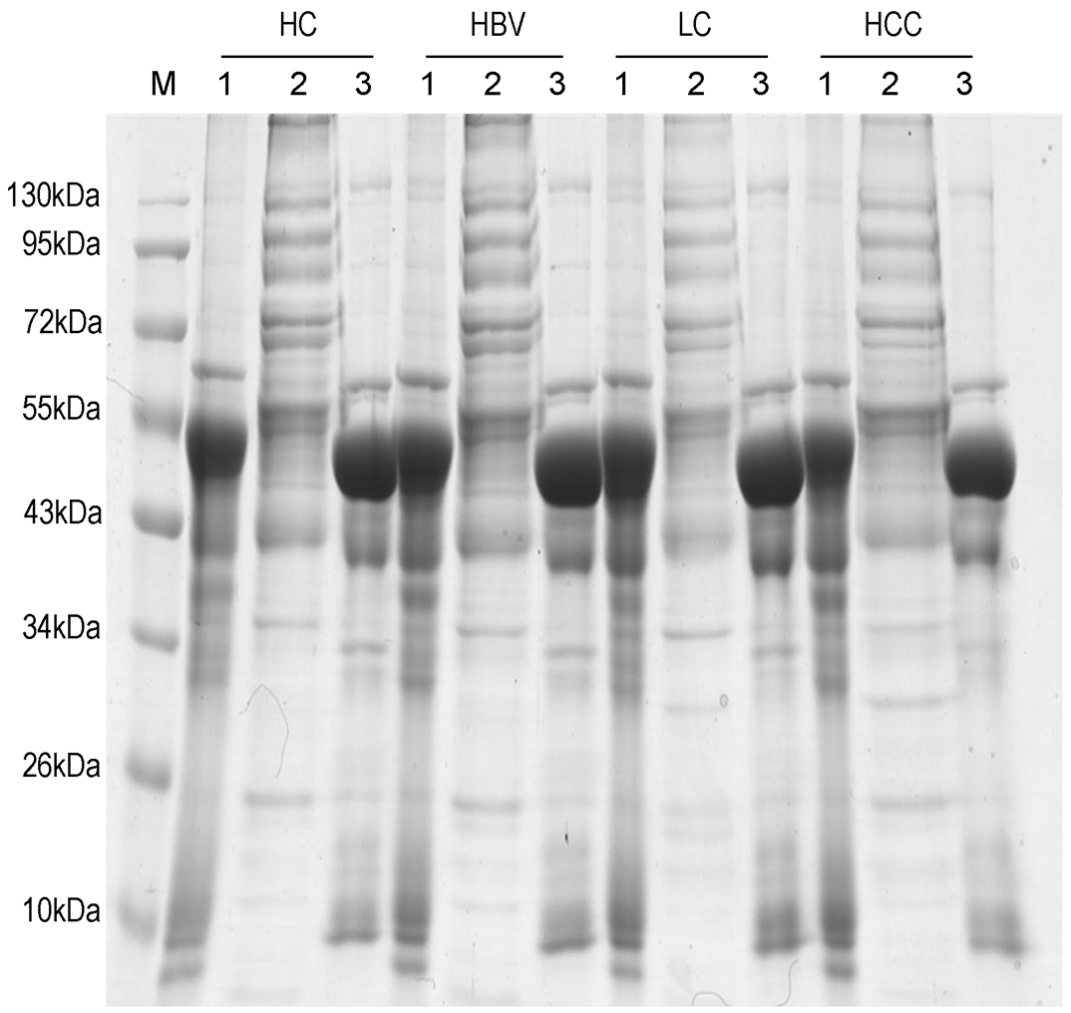
Sodium dodecyl sulfate polyacrylamide gel electrophoresis (SDS-PAGE) of serum after depletion of high-abundance proteins Lane M: molecular weight markers; lane 1: crude serum; lane 2: low-abundance proteins; lane 3: high-abundance proteins. HC: healthy controls; HBV: hepatitis B virus infection; LC: liver cirrhosis; HCC: hepatocellular carcinoma.

After MARS chromatography, pooled serum samples were separated into low- and high-abundance proteins. The following high-abundance proteins were largely eliminated: albumin, immunoglobulin G (IgG), antitrypsin, immunoglobulin A (IgA), transferrin, haptoglobin, fibrinogen, alpha2-macroglobulin, alpha1-acid glycoprotein, transthyretin, complement 3 (C3), apolipoprotein A-I (apoA-I), apolipoprotein A-II (apoA-II), and immunoglobulin M (IgM). Quantity One software analysis indicated that MARS removed 94% of high-abundance proteins, which significantly enriched the samples for low-abundance proteins (Figure 2).

### iTRAQ-LC-MS/MS analysis of serum proteins

**Figure 3:**
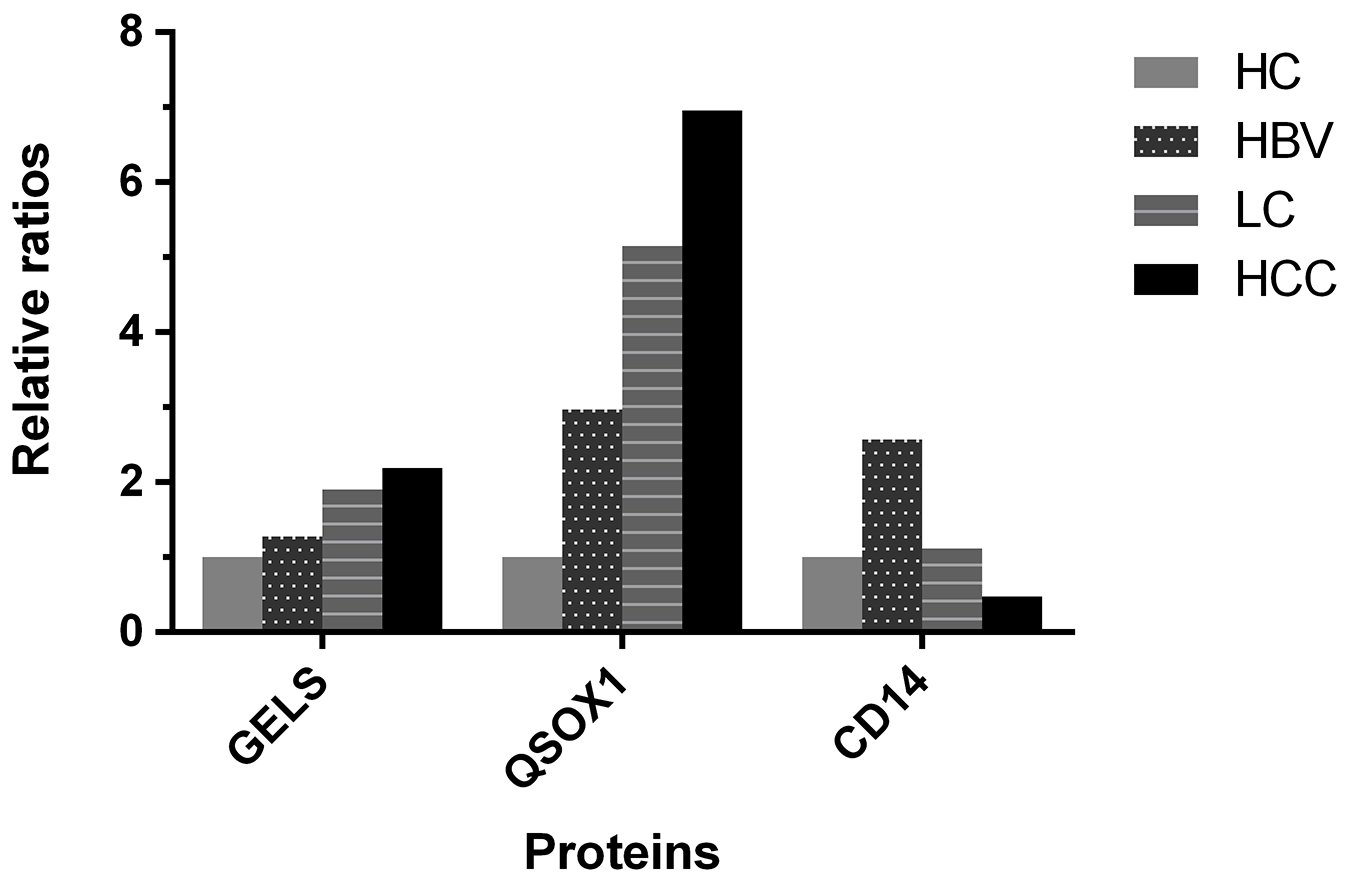
Relative ratios of three differentially expressed proteins (GELS, QSOX1 and CD14) compared with the HC group The protein was up-regulated with the relative ratio >1 and down-regulated with the relative ratio <1. GELS: gelsolin; QSOX1: sulfhydryl oxidase 1; HC: healthy controls; HBV: hepatitis B virus infection; LC: liver cirrhosis; HCC: hepatocellular carcinoma.

Of the 280 proteins analyzed in the discovery phase, 93 were found to be differentially expressed between at least two of the four participant groups. Relative to the HC group, 43 proteins were differentially expressed in the HBV group, 70 in the LC group and 51 in the HCC group. (Tables 2-5) We also identified several proteins that were differentially expressed among more than two groups. We chose to focus on these as potential HCC biomarkers because of the possibility that their expression level correlates with pathological stage of liver disease. We found that GELS was persistently up-regulated in more advanced disease: HBV / HC = 1.280 (*p*=0.028), LC / HBV = 1.489 (*p*=0.001), and HCC / LC = 1.150 (*p*=0.019). We observed a similar result with QSOX1: HBV / HC = 2.973 (*p*=0.016), LC / HBV = 1.733 (*p*=0.011), and HCC / LC = 1.350 (*p*=0.004). Conversely, CD14 was persistently down-regulated in more advanced disease: LC / HBV = 0.433 (*p*=0.035) and HCC / LC = 0.429 (*p*=0.019) (Figure 3)

**Table 2:** List of proteins differentially expressed between groups of hepatitis B virus infection and healthy controls

Accession	Protein name	iTRAQ ratio (HBV/HC)	*P*
**A2AP**	Alpha-2-antiplasmin	0.603	0.011
**A2MG**	Alpha-2-macroglobulin	6.982	<0.001
**AFAM**	Afamin	1.837	0.001
**AMBP**	Protein AMBP	0.391	0.036
**ANGT**	Angiotensinogen	0.387	0.027
**ANT3**	Antithrombin-III	0.545	<0.001
**APOA1**	Apolipoprotein A-I	2.754	<0.001
**APOA2**	Apolipoprotein A-II	3.499	0.019
**APOA4**	Apolipoprotein A-IV	1.459	0.013
**APOC1**	Apolipoprotein C-I	0.211	0.039
**APOC2**	Apolipoprotein C-II	2.109	0.010
**APOE**	Apolipoprotein E	0.34	0.004
**C1S**	Complement C1s subcomponent	1.786	0.018
**CERU**	Ceruloplasmin	0.581	<0.001
**CFAH**	Complement factor H	1.803	0.010
**CFAI**	Complement factor I	1.472	0.018
**CO3**	Complement C3	0.565	0.025
**CO7**	Complement component C7	1.706	0.025
**FCN3**	Ficolin-3	2.128	0.027
**FETUA**	Alpha-2-HS-glycoprotein	3.664	0.013
**HBA**	Hemoglobin subunit alpha	0.331	0.006
**HBB**	Hemoglobin subunit beta	0.337	0.016
**HPT**	Haptoglobin	0.56	<0.001
**HRG**	Histidine-rich glycoprotein	0.356	0.020
**IC1**	Plasma protease C1 inhibitor	0.667	0.048
**IGHA1**	Ig alpha-1 chain C region	5.105	0.044
**IGHG3**	Ig gamma-3 chain C region	4.966	<0.001
**IGHM**	Ig mu chain C region	7.656	<0.001
**IGKC**	Ig kappa chain C region	6.368	0.004
**IGLL5**	Immunoglobulin lambda-like polypeptide 5	2.831	0.039
**ITIH1**	Inter-alpha-trypsin inhibitor heavychain H1	0.603	0.008
**ITIH2**	Inter-alpha-trypsin inhibitor heavychain H2	0.655	0.009
**K1C9**	Keratin, type I cytoskeletal 9	4.207	0.003
**K22E**	Keratin, type II cytoskeletal 2 epidermal	3.373	0.030
**K2C1**	Keratin, type II cytoskeletal 1	4.613	0.001
**LUM**	Lumican	1.803	0.037
**NRP1**	Neuropilin-1	2.535	0.003
**PEDF**	Pigment epithelium-derived factor	1.57	0.020
**RET4**	Retinol-binding protein 4	0.506	0.020
**THRB**	Prothrombin	1.837	0.048
**TRFE**	Serotransferrin	1.923	0.002
**VTDB**	Vitamin D-binding protein	0.586	0.012
**ZA2G**	Zinc-alpha-2-glycoprotein	0.466	0.028

HBV, hepatitis B virus infection; HC, healthy controls.

**Table 3:** List of proteins differentially expressed between groups of liver cirrhosis and healthy controls

Accession	Protein name	iTRAQ ratio (LC/HC)	*P*
**A1AG2**	Alpha-1-acid glycoprotein 2	0.256	0.031
**A1AT**	Alpha-1-antitrypsin	1.624	0.001
**A1BG**	Alpha-1B-glycoprotein	2.128	0.001
**A2AP**	Alpha-2-antiplasmin	0.296	0.005
**A2GL**	Leucine-rich alpha-2-glycoprotein	1.514	0.036
**A2MG**	Alpha-2-macroglobulin	5.2	<0.001
**AACT**	Alpha-1-antichymotrypsin	0.433	0.025
**AFAM**	Afamin	1.722	0.024
**ALS**	Insulin-like growth factor-binding protein complex acid labile subunit	0.322	<0.001
**AMBP**	Protein AMBP	0.347	0.027
**ANGT**	Angiotensinogen	0.38	0.015
**ANT3**	Antithrombin-III	0.377	<0.001
**APOA1**	Apolipoprotein A-I	0.466	0.031
**APOA2**	Apolipoprotein A-II	0.203	0.045
**APOA4**	Apolipoprotein A-IV	2.831	<0.001
**APOM**	Apolipoprotein M	0.166	0.044
**C1QA**	Complement C1q subcomponent subunit A	1.977	0.010
**C1QB**	Complement C1q subcomponent subunit B	3.133	0.003
**C1R**	Complement C1r subcomponent	0.337	0.048
**C4BPA**	C4b-binding protein alpha chain	0.27	0.026
**CD5L**	CD5 antigen-like	3.342	0.012
**CERU**	Ceruloplasmin	0.337	<0.001
**CFAB**	Complement factor B	0.322	<0.001
**CFAH**	Complement factor H	0.225	<0.001
**CFAI**	Complement factor I	0.296	0.001
**CNDP1**	Beta-Ala-His dipeptidase	0.225	0.032
**CO2**	Complement C2	0.334	0.004
**CO3**	Complement C3	4.571	0.002
**CO5**	Complement C5	0.429	<0.001
**CO6**	Complement component C6	0.429	0.039
**CO7**	Complement component C7	6.73	<0.001
**CXCL7**	Platelet basic protein	0.127	0.003
**F13B**	Coagulation factor XIII B chain	0.592	0.028
**FA5**	Coagulation factor V	0.217	0.032
**FETUA**	Alpha-2-HS-glycoprotein	3.467	<0.001
**FINC**	Fibronectin	0.592	0.001
**GELS**	Gelsolin	1.355	0.029
**HBA**	Hemoglobin subunit alpha	0.242	0.027
**HEMO**	Hemopexin	0.278	<0.001
**HEP2**	Heparin cofactor 2	0.406	0.002
**HPT**	Haptoglobin	0.251	<0.001
**IC1**	Plasma protease C1 inhibitor	0.637	<0.001
**IGHG3**	Ig gamma-3 chain C region	7.516	<0.001
**IGHM**	Ig mu chain C region	5.395	0.005
**IGKC**	Ig kappa chain C region	10.375	0.001
**IPSP**	Plasma serine protease inhibitor	2.704	0.035
**ITIH1**	Inter-alpha-trypsin inhibitor heavy chain H1	0.278	<0.001
**ITIH2**	Inter-alpha-trypsin inhibitor heavy chain H2	0.344	<0.001
**ITIH4**	Inter-alpha-trypsin inhibitor heavy chain H4	0.525	0.022
**K2C1**	Keratin, type II cytoskeletal 1	4.831	0.011
**KAIN**	Kallistatin	0.429	0.038
**KLKB1**	Plasma kallikrein	0.273	<0.001
**KNG1**	Kininogen-1	0.319	0.002
**KV313**	Ig kappa chain V-III region HIC	3.048	0.025
**LUM**	Lumican	2.655	<0.001
**PEDF**	Pigment epithelium-derived factor	1.905	0.024
**PLMN**	Plasminogen	0.256	<0.001
**PRG4**	Proteoglycan 4	0.24	0.021
**PROS**	Vitamin K-dependent protein S	0.244	0.035
**QSOX1**	Sulfhydryl oxidase 1	7.447	0.020
**RET4**	Retinol-binding protein 4	0.421	0.019
**SAMP**	Serum amyloid P-component	0.143	0.003
**SEPP1**	Selenoprotein P	2.355	0.018
**THRB**	Prothrombin	0.479	0.032
**TRFE**	Serotransferrin	0.479	<0.001
**TSP1**	Thrombospondin-1	0.141	<0.001
**VTDB**	Vitamin D-binding protein	0.425	<0.001
**VTNC**	Vitronectin	0.283	0.002
**VWF**	von Willebrand factor	6.31	0.001
**ZPI**	Protein Z-dependent protease inhibitor	0.402	0.002

LC, liver cirrhosis; HC, healthy controls.

**Table 4:** List of proteins differentially expressed between groups of hepatocellular carcinoma and healthy controls

Accession	Name	iTRAQ ratio (HCC/HC)	*P*
**A1AG2**	Alpha-1-acid glycoprotein 2	0.57	0.022
**A1AT**	Alpha-1-antitrypsin	1.995	0.002
**A1BG**	Alpha-1B-glycoprotein	0.667	0.032
**A2AP**	Alpha-2-antiplasmin	0.752	0.037
**A2MG**	Alpha-2-macroglobulin	2.032	<0.001
**AACT**	Alpha-1-antichymotrypsin	0.731	0.041
**ACTG**	Actin, cytoplasmic 2	2.148	0.001
**AFAM**	Afamin	3.162	<0.001
**ALS**	Insulin-like growth factor-binding protein complex acid labile subunit	0.344	0.001
**ANT3**	Antithrombin-III	0.363	<0.001
**APOA2**	Apolipoprotein A-II	14.723	<0.001
**APOA4**	Apolipoprotein A-IV	0.347	<0.001
**C1S**	Complement C1s subcomponent	0.619	0.026
**CBG**	Corticosteroid-binding globulin	0.492	0.021
**CBPN**	Carboxypeptidase N catalytic chain	2.704	0.021
**CO2**	Complement C2	2.831	0.031
**CO3**	Complement C3	9.727	<0.001
**CO5**	Complement C5	1.629	0.029
**CO7**	Complement component C7	2.535	0.005
**CO9**	Complement component C9	2.399	0.037
**CPN2**	Carboxypeptidase N subunit 2	2.858	0.009
**F13B**	Coagulation factor XIII B chain	0.353	0.008
**FETA**	Alpha-fetoprotein	33.113	0.009
**FETUA**	Alpha-2-HS-glycoprotein	0.291	0.026
**FINC**	Fibronectin	0.238	<0.001
**GELS**	Gelsolin	2.938	<0.001
**HBA**	Hemoglobin subunit alpha	1.528	0.047
**HBB**	Hemoglobin subunit beta	0.291	0.011
**HEP2**	Heparin cofactor 2	0.555	0.010
**HPT**	Haptoglobin	0.158	<0.001
**IC1**	Plasma protease C1 inhibitor	0.219	<0.001
**IGHM**	Ig mu chain C region	2.109	0.002
**IGKC**	Ig kappa chain C region	9.727	0.001
**ITIH1**	Inter-alpha-trypsin inhibitor heavy chain H1	0.409	0.003
**ITIH2**	Inter-alpha-trypsin inhibitor heavy chain H2	0.466	<0.001
**K1C10**	Keratin, type I cytoskeletal 10	11.272	0.035
**K1C9**	Keratin, type I cytoskeletal 9	4.207	0.001
**K2C1**	Keratin, type II cytoskeletal 1	2.729	0.019
**K2C6C**	Keratin, type II cytoskeletal 6C	6.31	<0.001
**KLKB1**	Plasma kallikrein	0.57	0.010
**KNG1**	Kininogen-1	0.673	0.007
**KV313**	Ig kappa chain V-III region HIC	3.532	0.016
**LBP**	Lipopolysaccharide-binding protein	5.058	0.001
**LUM**	Lumican	2.559	0.004
**PGRP2**	N-acetylmuramoyl-L-alanine amidase	0.296	0.029
**RET4**	Retinol-binding protein 4	0.347	0.003
**TRFE**	Serotransferrin	0.174	<0.001
**VTDB**	Vitamin D-binding protein	0.515	0.001
**VTNC**	Vitronectin	0.575	0.015
**VWF**	von Willebrand factor	4.446	0.008
**ZA2G**	Zinc-alpha-2-glycoprotein	0.625	0.012

HCC, hepatocellular carcinoma; HC, healthy controls.

**Table 5:** List of proteins identified as differentially expressed both in the present study and in previous work

Accession	Protein name	Expression trend	Reference
**AFP**	Alpha-fetoprotein	↑	Li 2009 [18]
**CERU**	Ceruloplasmin	↑	Feng 2005 [16]
**A1AT**	Alpha-1-antitrypsin	↑	Qin 2013 [20]
			Feng 2005 [16]
**APOE**	Apolipoprotein E	↑	Blanc 2005 [15]
			Zhong 2012 [23]
**APOA2**	Apolipoprotein A-II	↑	Zhong 2012 [23]
**PON1**	Serum paraoxonase/arylesterase 1	↑	Huang 2013 [17]
**ACTG**	Actin, cytoplasmic 2	↑	Xu 2013 [22]
**C3**	Complement C3	↑	Steel 2003 [21]
**APOA1**	Apolipoprotein A-I	↓	Qin 2013 [20]
			Zhong 2012 [23]
			Steel 2003 [21]
**IPSP**	Plasma serine protease inhibitor	↓	Marshall 2013 [19]

↑ Up-regulated; ↓ Down-regulated.

### Bioinformatic analysis

**Figure 4:**
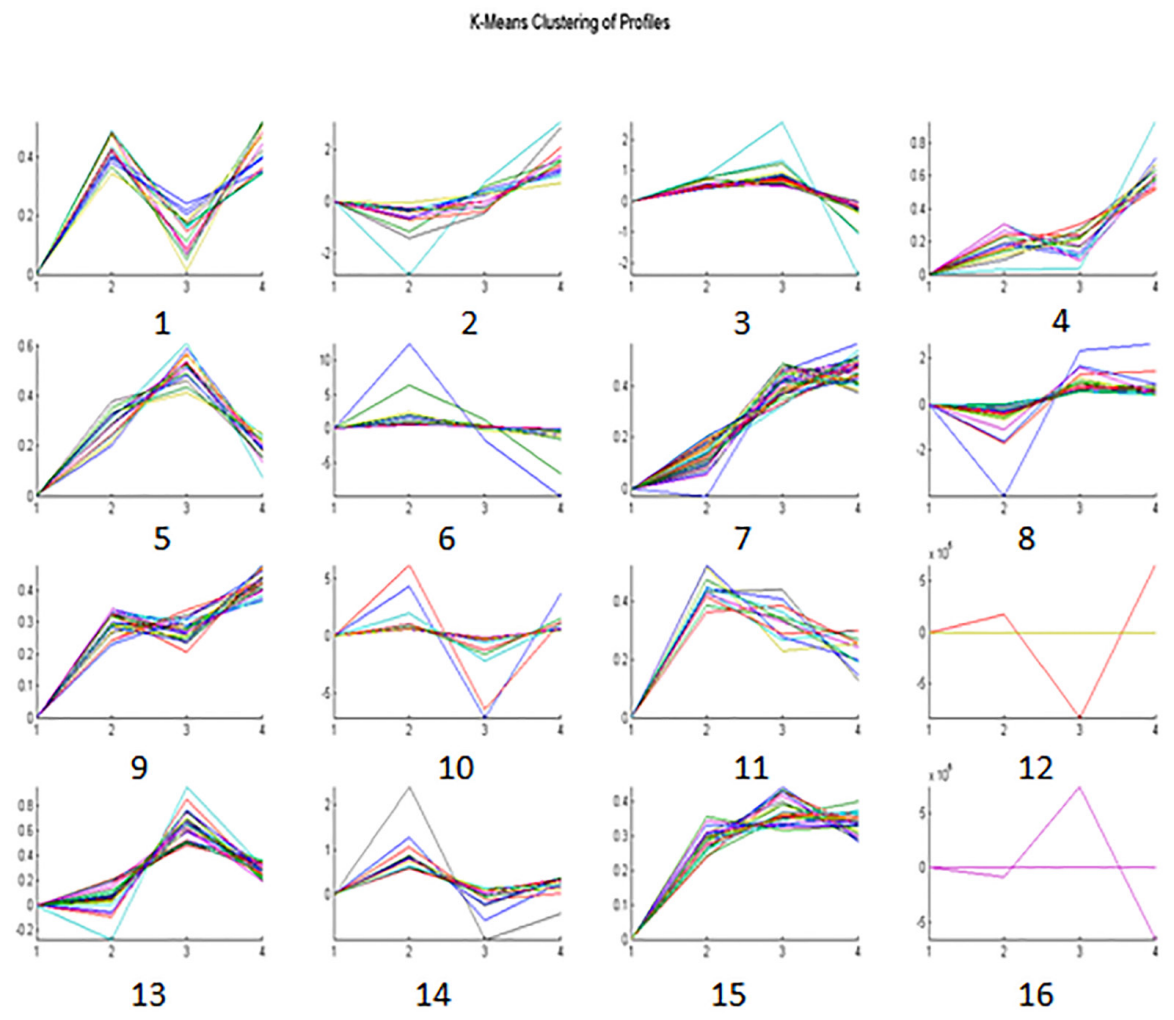
K-means cluster analysis of differentially expressed proteins The abscissa represents the four groups: 1 (HC), 2 (HBV), 3 (LC) and 4 (HCC). The ordinate represents the expression patterns of the differentially expressed proteins. HC: healthy controls; HBV: hepatitis B virus infection; LC: liver cirrhosis; HCC: hepatocellular carcinoma.

**Figure 5:**
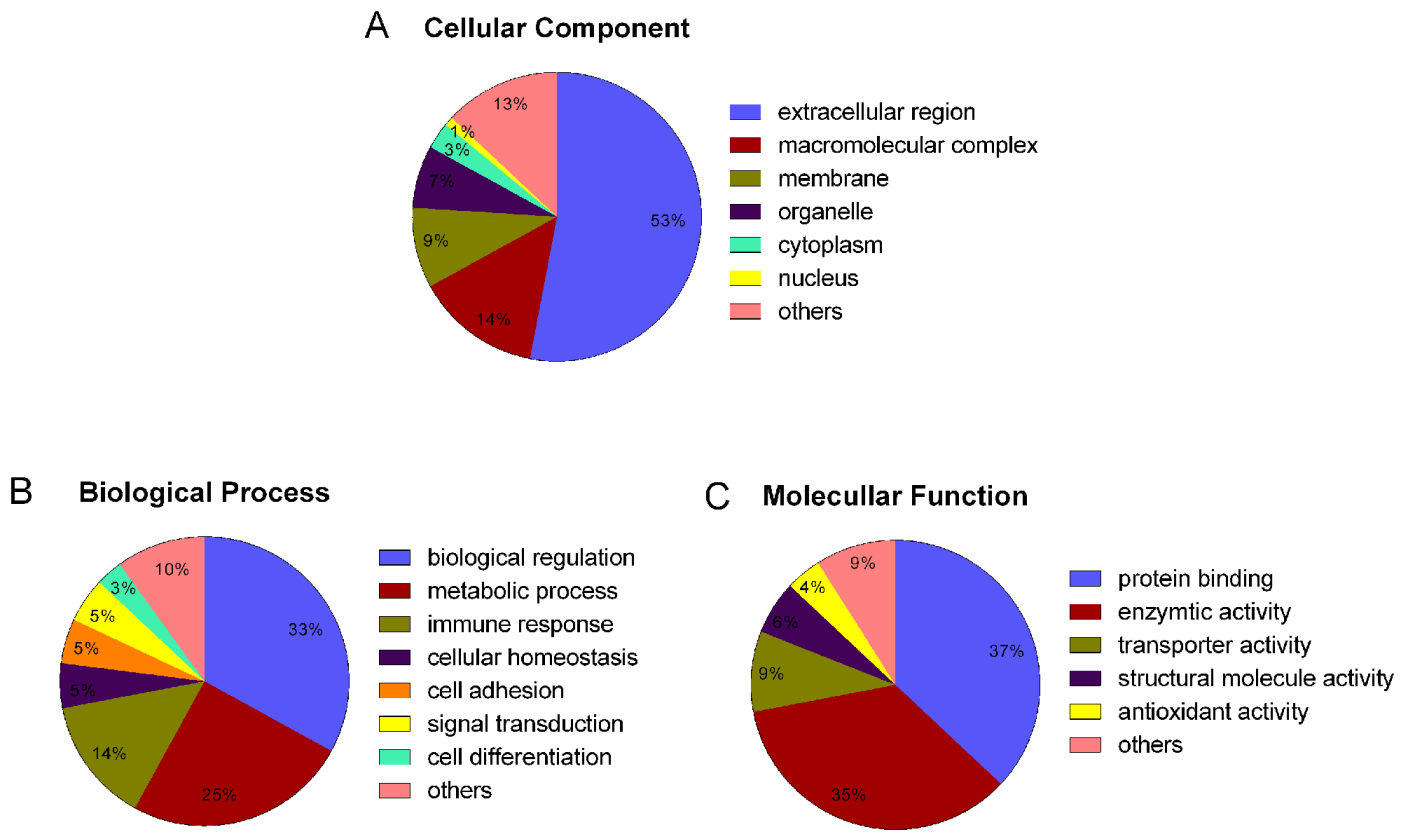
Gene ontology classification of differentially expressed proteins based on their cellular localization, biological process and molecular function **(A)** Distribution of proteins based on their cellular localization. **(B)** Distribution of proteins based on their biological process. **(C)** Distribution of proteins based on their molecular function.

**Figure 6:**
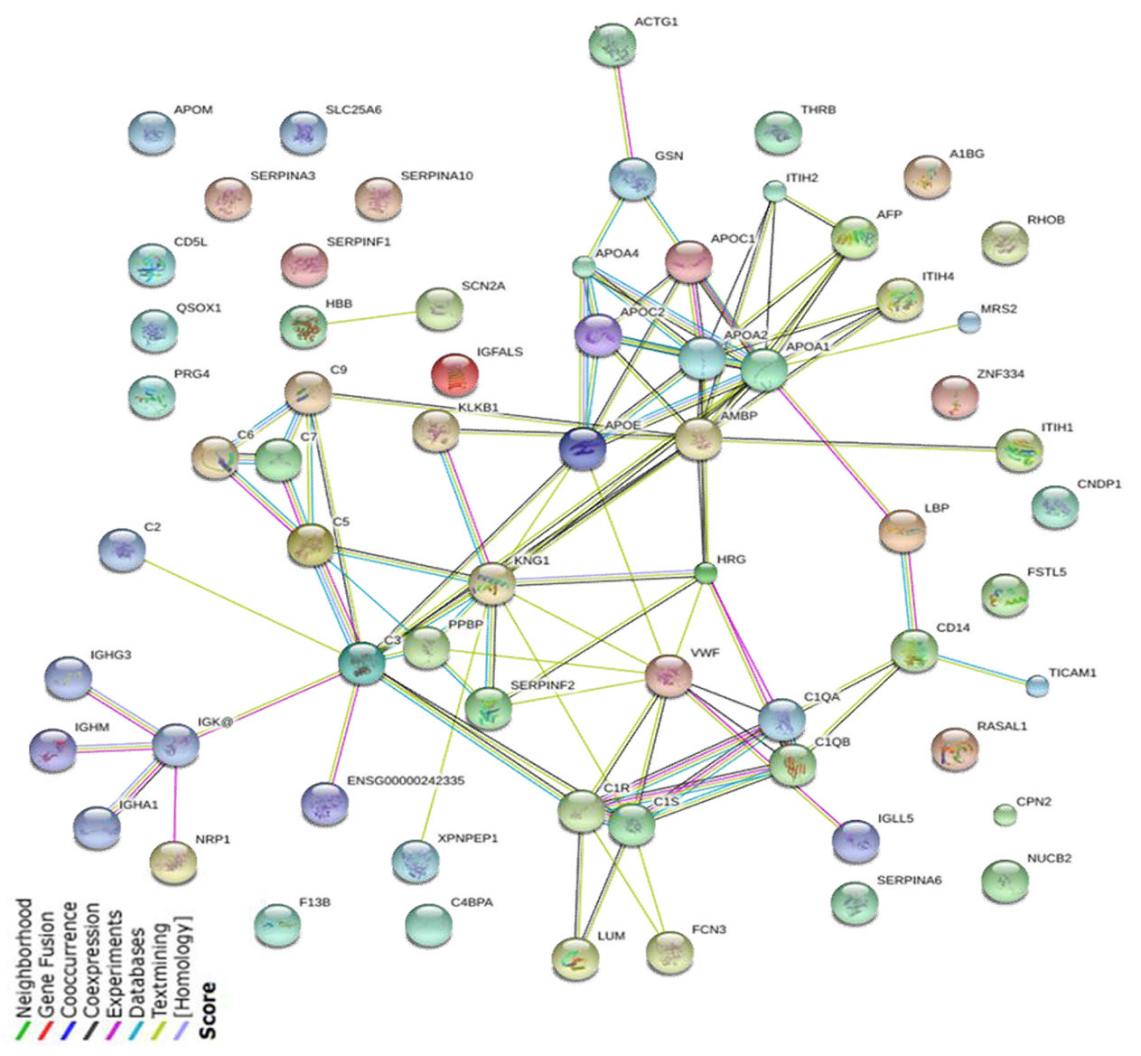
String network analysis of differentially expressed proteins identified by iTRAQ-LC-MS/MS Lines with different colors represent the existence of different types of evidence applied to predict associations between proteins.

Bioinformatics was used to classify proteins on the basis of change trends, biological process, molecular function and subcellular localization. Figure 4 summarizes the tendency patterns for differentially expressed proteins. A total of 36 types of K-means patterns were acquired: patterns 4 and 34 showed a trend of increasing protein expression under HCC conditions, while patterns 2, 9 and 19 showed a trend of increasing expression under HBV conditions, followed by a trend of decreasing expression under LC and HCC conditions. Further analysis showed that proteins in patterns 4 and 34 were involved mainly in metabolism, while proteins in patterns 2, 9 and 19 were mainly complementary proteins. GO analysis suggested that proteins were located mainly in the extracellular matrix (53%) and they participated mainly in biological regulation (33%), metabolism (25%) and immune responses (14%). Molecular functions were mainly protein binding (37%) and enzymatic activity (35%) (Figure 5). String network analysis identified various possible interactions among the proteins, with several linked to more than 4 interactors (Figure 6).

### Validation of CD14, GELS and QSOX1 by Western blot

**Figure 7:**
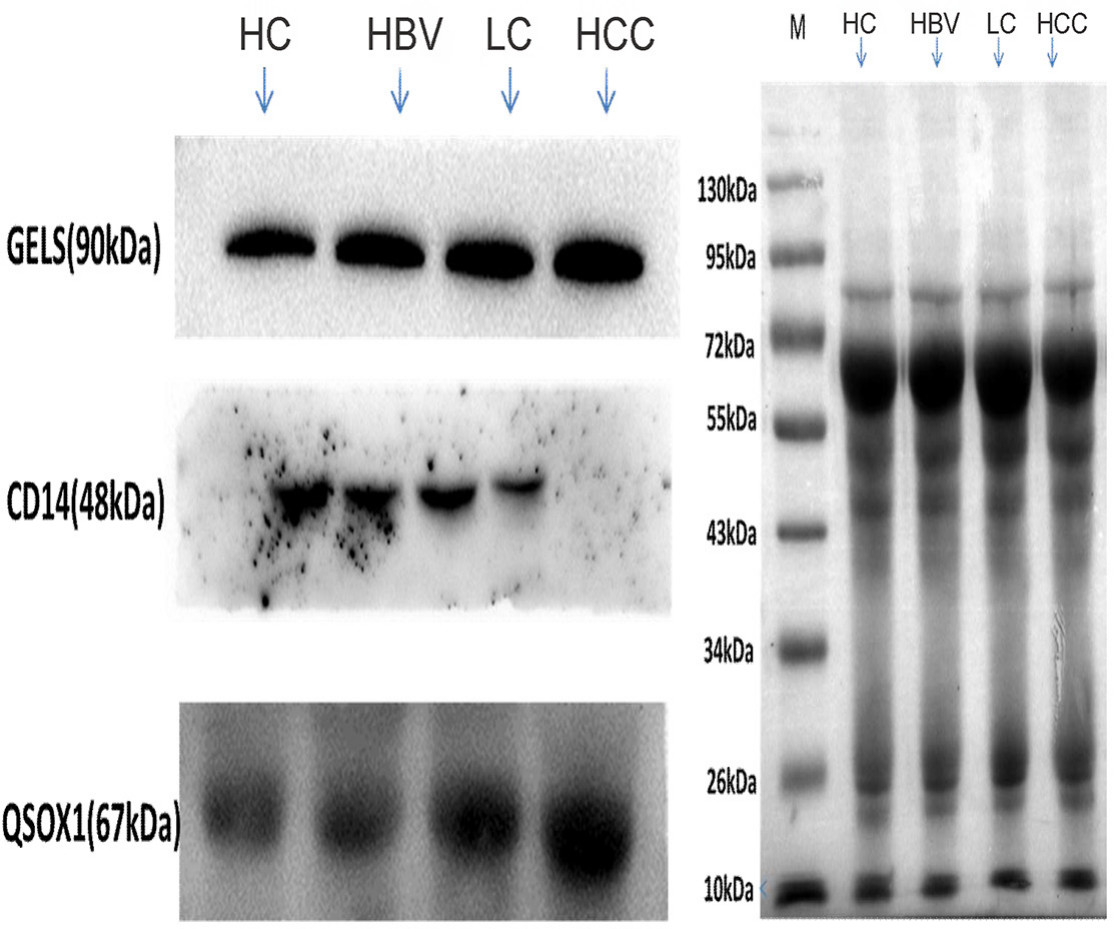
Validation of three differentially expressed proteins (GELS, QSOX1 and CD14) by Western blot analysis The grey value of the serum protein stripe, which was measured after staining the PVDF membrane with MemCode, was used as an internal control. GELS: gelsolin; QSOX1: sulfhydryl oxidase 1; HC: healthy controls; HBV: hepatitis B virus infection; LC: liver cirrhosis; HCC: hepatocellular carcinoma.

CD14, GELS and QSOX1 were chosen for further validation. The main reason was that their expression difference was more obvious among different groups and the consistent up- or down-regulation observed may indicate a relatively stable and continuous function in disease progression. Consistent with the MS analysis, Western blotting showed progressive up-regulation of GELS and QSOX1 with more advanced liver disease, and progressive down-regulation of CD14 with more advanced disease (Figure 7).

### Quantification of CD14 by ELISA

**Figure 8:**
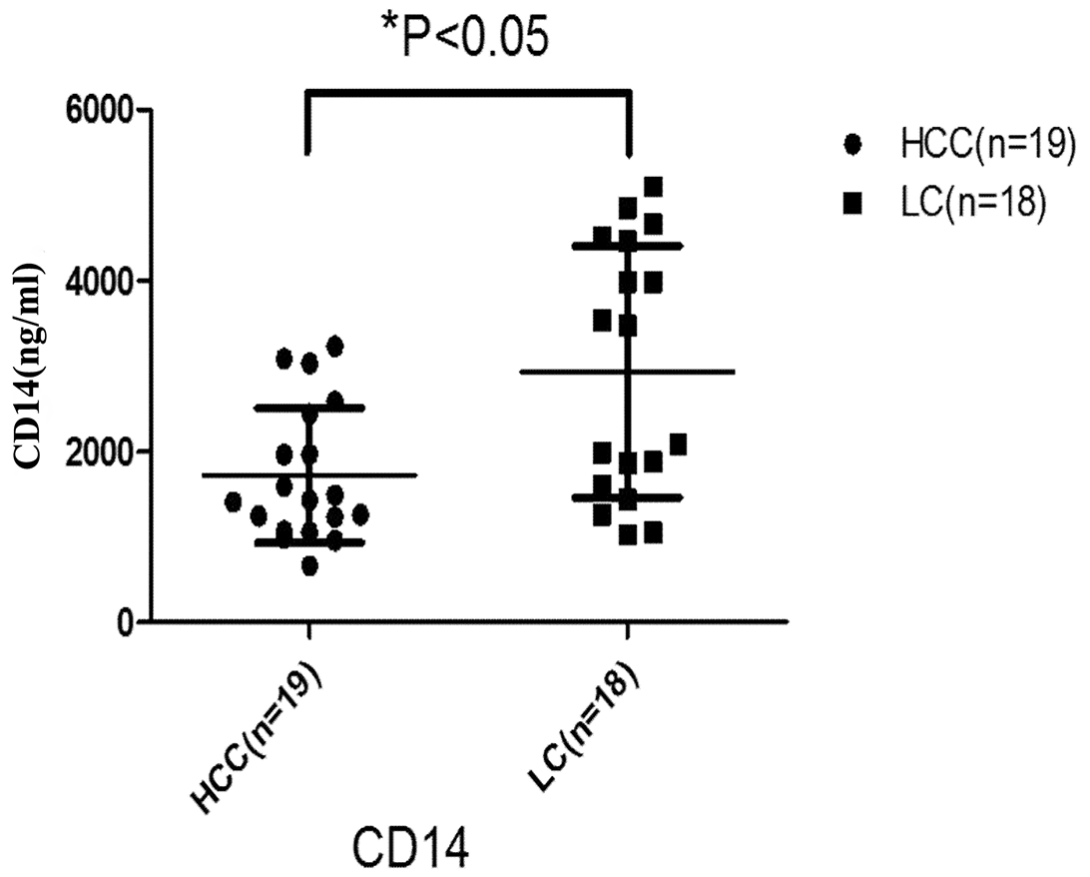
Serum concentration of CD14 in HCC and LC groups The LC group showed a significantly higher level (p = 0.003). Black horizontal lines are means, and error bars are SEs. LC: liver cirrhosis; HCC: hepatocellular carcinoma.

As a further validation of the biomarker screening, we used ELISA to measure CD14 levels in HCC and LC serum samples. Levels were compared using the approximate *t* test because of the heterogeneity of sample variance. CD14 levels were significantly lower in the HCC samples (F *=* 22.487, *p* = 0.005; Figure 8), consistent with the MS analysis.

### Logistic regression and ROC curve analysis

**Figure 9:**
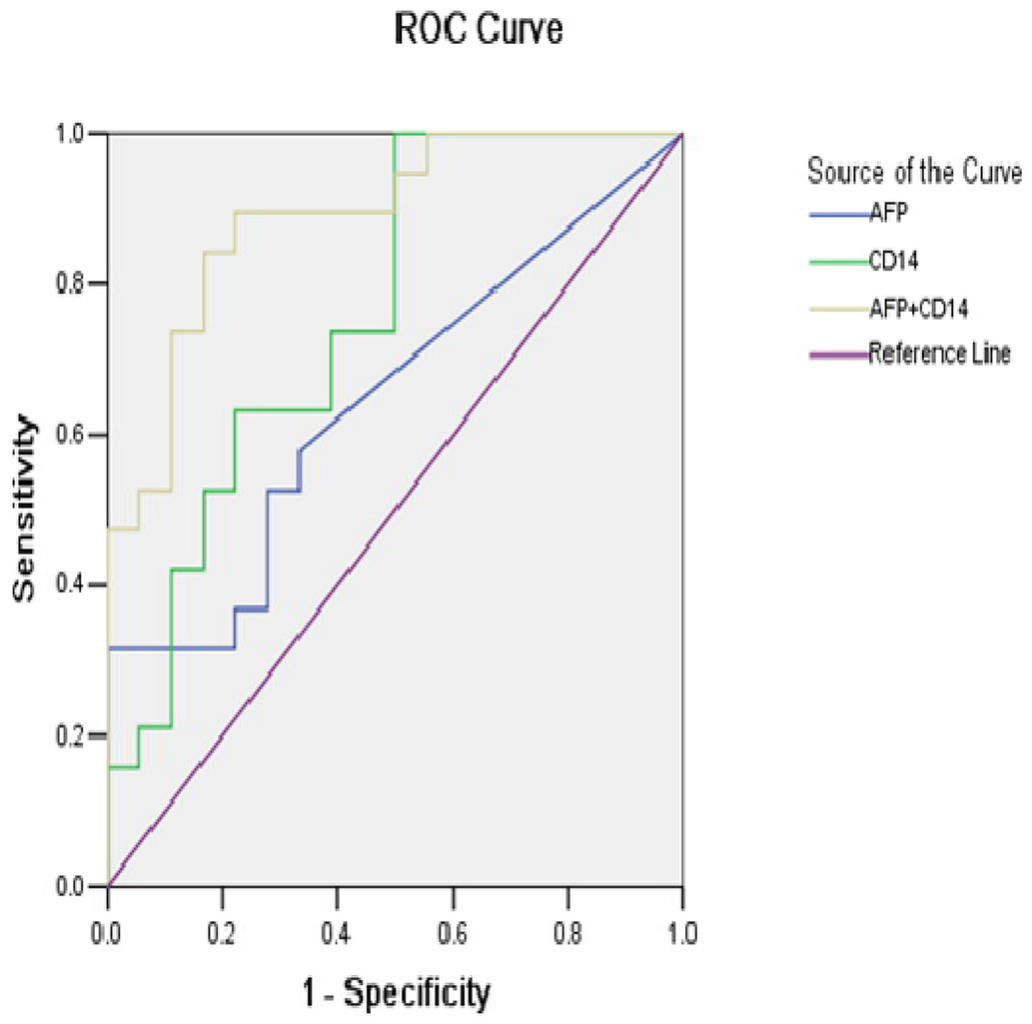
Receiver operating characteristic (ROC) curves for AFP alone, CD14 alone and their combination in the diagnosis of HCC AUC: area under ROC curve; AFP: alpha-fetoprotein; HCC: hepatocellular carcinoma.

The logistic equation was as follows: ln [p/(1–p)] = –2.532 – 0.002 × CD14 – 0.016 × AFP, where p refers to the probability of HCC. Receiver operating characteristic (ROC) curves were generated to validate the diagnostic value of the model (Figure 9). The results indicated that the optimal diagnostic cut-off for AFP was 191.4 ng/mL [area under the curve (AUC) 0.646, 95%CI 0.467-0.825, sensitivity 31.6%, specificity 94.4%), the optimal cut-off for CD14 was 3.16 ng/mL (AUC 0.760, 95%CI 0.604-0.917, sensitivity 94.7%, specificity 50%), and the optimal cut-off for the combination of AFP and CD14 was 0.197 ng/mL (AUC 0.889, 95%CI 0.785-0.993, sensitivity 84.2%, specificity 83.3%). Logistic regression suggested that for the combination of the two serum biomarkers, overall accuracy was 81.1%; positive predictive value, 89.5%; and negative predictive value, 72.2%.

## DISCUSSION

HCC, the most common histological type of liver neoplasms, is a highly lethal disease difficult to diagnose early and therefore treat aggressively. HCC carcinogenesis is known to be a multistage process often involving HBV infection and liver cirrhosis. Therefore we screened individuals at different stages of liver disease in order to capture dynamic changes in the serum proteome that might lead to the identification of novel serum biomarkers for detecting early HCC. Using high-throughput iTRAQ quantitative proteomics and a large-cohort serum bank, we identified 93 proteins differentially expressed between healthy controls and individuals with different stages of liver disease. And further we selected CD14, QSOX1 and GELS that were differentially expressed among the four groups for validation by Western blot or ELISA.

Functions are unknown for most of the differentially expressed proteins identified in our study. GO analysis suggests that most of these proteins are secreted and are involved in biological processes. K-means analysis identified 36 patterns, and string network analysis indicated various interactions among the proteins. Several of them, including AFP, apoA-I, apolipoprotein E (apoE), GELS, CD14, C3 and inter-alpha inhibitor H4 (ITIH4), mapped over four proteins in the network diagrams, and thus may play crucial roles in HCC detection. The reliability of our results is reflected in the fact that several proteins identified in our study as significantly up- or down-regulated in liver disease were reported to show a similar relationship in previous studies using a different methodology. In particular, AFP, C3, apoA-III, apoE, alpha-1-antitrypsin (A1AT), ACTG (actin, cytoplasmic 2), ceruloplasmin (CERU) and serum paraoxonase/arylesterase 1 (PON1) were previously reported to be up-regulated as in our study, while apoA-I, serum amyloid P-component (SAMP), plasma serine protease inhibitor (IPSP) were reported to be down-regulated [15-23].

The difficulty of early diagnosing HCC is mainly attributed to the current serum biomarkers could not effectively distinguish HCC from LC. Thus in the validation phase of our study, we only recruited HCC and LC patients. The result indicated that AFP was unable to distinguish HCC from LC. Approximately one third of HCC patients do not express AFP, while it is expressed in just 20-60% of HBV and LC patients, [24] making it a less reliable HCC biomarker. In our study, CD14 was able to distinguish HCC from LC (p < 0.05), so it may be a complementary biomarker for AFP in HCC diagnosis. Indeed, combining AFP and CD14 improved the diagnosis rate of HCC, increasing sensitivity to 84.2% and specificity to 83.3%. CD14 is a glycoprotein comprising a membrane-bound part (mCD14) and a soluble part (sCD14). The mCD14 is mainly distributed on the surface of mononuclear cells, macrophages and dendritic cells, while sCD14 is secreted by mononuclear cells and exists in the serum of human and animals. Our Western and ELISA assays specifically recognized sCD14. This molecule appears to bind the bacterial endotoxin lipopolysaccharide (LPS) and help trigger host immune responses against the bacterial invader [25].

The quantitative iTRAQ method has been used to investigate candidate biomarkers for a number of tumors, including oral cancer, [26] renal cell carcinoma, [27] breast cancer, [28] lung cancer, [29] ovarian cancer [30] as well as nasopharyngeal carcinoma [31]. While it has also been applied to the study of HCC, [17, 32-35] we are aware of only one study focusing on HCC diagnosis, and the authors employed mainly cell lines [32]. The present study relied on serum samples from patients specifically recruited because their liver disease was prior to HCC or in early stages of HCC. Samples came from a large cohort of HBsAg-positive individuals at high risk of liver cancer who were living in an area of China with endemic liver cancer. These individuals have been followed up and their serum sampled regularly since 2003. This specimen bank and database is uniquely suited to allowing analysis of serum proteins that may correlate with HCC onset and progression. The present work represents a substantial first step in this direction.

Furthermore, all the patients with HCC in our study recruited from this specimen bank had cirrhosis and all HBV patients received no treatments. Thus, we can rule out the impact of these factors. However, the relatively small sample size enrolled in the verification phase may reduce the reliability of the conclusion. Therefore, further studies of large cohorts of patients are needed to investigate the functions of the differentially expressed proteins we identified and further validate the diagnostic value of CD14 alone or in combination with other biomarkers such as AFP. The discovery approach here, and the serum specimen bank on which it is based, may be useful for detecting HCC early enough to allow aggressive, curative surgery to become an option for more patients.

## MATERIALS AND METHODS

### Ethics statement

This work was approved by the Research Ethics Committee of the Affiliated Tumor Hospital of Guangxi Medical University. Patient health, safety and privacy were protected throughout the study; serum and data collection were maintained anonymous. Written informed consent of all participants was obtained before the study began.

### Patients and serum samples

The study involved consecutive series of patients with HCC (HCC group), liver cirrhosis (LC group), or chronic hepatitis B virus (HBV) infection recruited from the specimen bank which was established based on the serum samples of high incidence area of HCC in Fusui, Guangxi, China, between October 2010 and May 2012. In parallel, completely healthy controls (population-based HC group) from blood donor were also recruited. During the discovery phase using iTRAQ, 40 participants were analyzed, 10 from each group. During the validation phase, 19 HCC patients and 18 LC patients were enrolled. Serum samples were collected from the specimen bank between October 2010 and May 2012 and stored at -80 °C until further analysis.

Primary HCC was diagnosed on the basis of ultrasound characteristics, serum level of alpha-fetoprotein (AFP), and histopathology results provided by the Affiliated Tumor Hospital of Guangxi Medical University in Guangxi, China. Diagnosis was carried out according to the guidelines of the American Association for the Study of Liver Diseases [36]. HBV infection was diagnosed based on the presence of HBsAg for 6 months prior to serum sampling, in accord with guidelines for preventing and treating chronic HBV infection [37]. Liver cirrhosis was diagnosed on the basis of histopathology of liver biopsy samples.

### Enrichment of low-abundance proteins

Serum biomarkers of disease are expected to be low-abundance proteins, which means they can be masked by high-abundance proteins that account for approximately 90% of all serum proteins. In order to enrich for low-abundance proteins, [38] high-abundance proteins were depleted using the Agilent multiple affinity removal LC column-Human 14 (MARS, 4.6 × 50 mm, Agilent Technologies, Santa Clara, CA, USA) according to the manufacturer’s instructions. In brief, serum samples were fractionated on MARS, and fractions were analyzed by SDS-PAGE and Coomassie blue staining to confirm the efficient removal of high-abundance proteins. Then relative band intensities were compared between different disease or control groups using Quantity One software (Bio-rad, USA).

### iTRAQ labeling

Serum samples were mixed and dialyzed against dissolution buffer using spin concentrators with a 5-kDa molecular weight cut-off (Millipore Technologies, USA), then concentrated to approximately 50 ml. Proteins were deoxidized using tris (2-carboxyethyl) phosphine (TCEP), blocked using S-methyl methanethiosulfonate (MMTS), digested with trypsin, and finally labeled with 8-plex iTRAQ reagents. The HC sample was labeled with reagent 113; HBV sample, reagent 114; LC sample, reagent 115; and HCC sample, reagent 116. In a second run, these samples were labeled with, respectively, reagents 117, 118, 119, and 121.

### LC-MS/MS analysis

The iTRAQ-labeled serum samples were pooled and desalted on a C18 column (Sep-Pak vac, 1 cc, 50 mg, Waters, Germany). The desalted mixtures were diluted in buffer A (5% acetonitrile, 94.5% water, 0.1% trifluoroacetic acid) and fractionated on a ZORBAX 300SB-C18 Column (5μm, 300A, 0.5×23mm, Waters, Germany). connected to a 20AD HPLC system (Shimadzu, Kyoto, Japan). After chromatographic separation for 70 min, the sample was eluted by applying a linear gradient of buffer B (95% acetonitrile, 4.99% water, 0.1% trifluoroacetic acid) from 5% to 35% over 53 min. Fractions were collected every 2 min, giving a total of 35 fractions.

**Figure 10:**
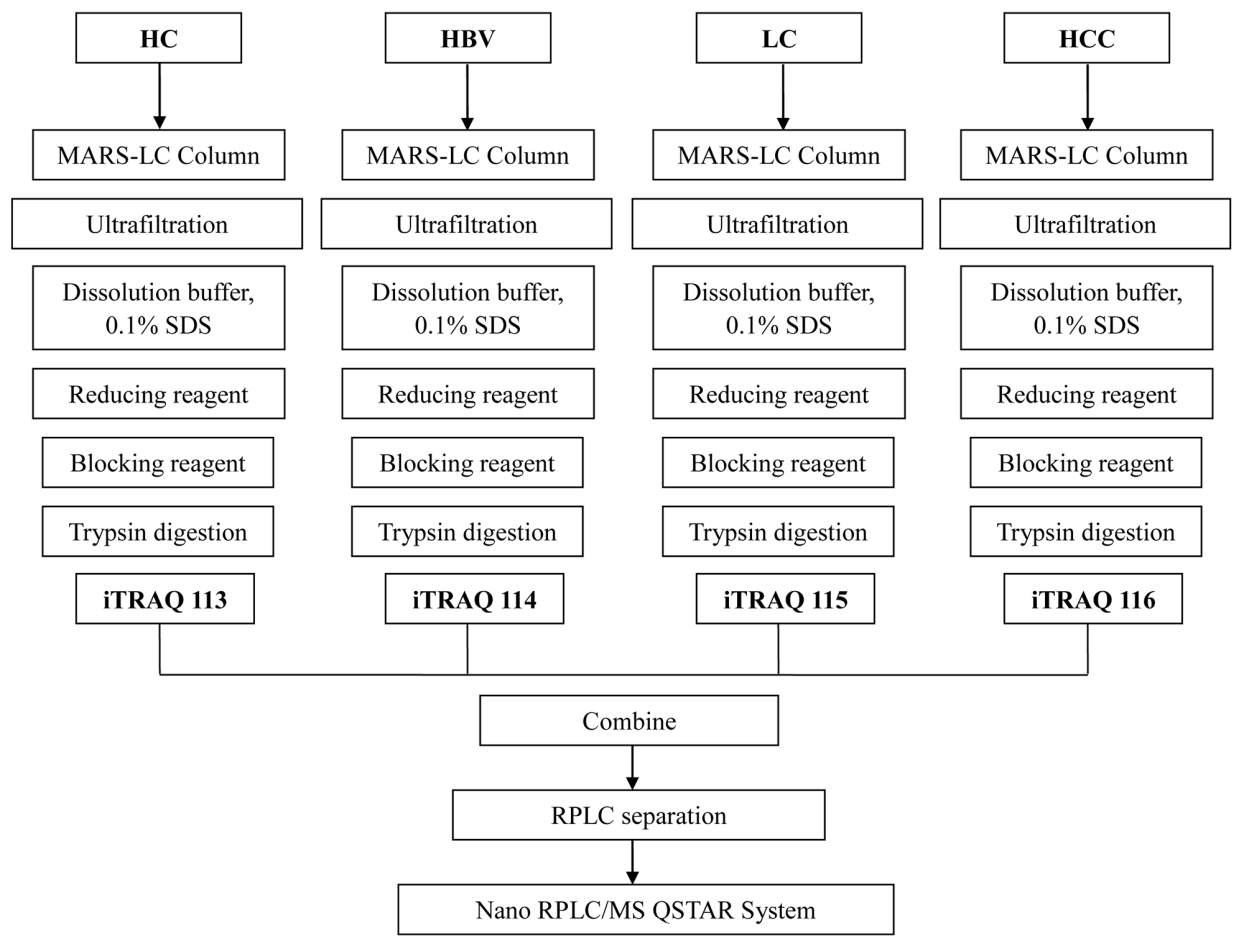
Flow diagram describing the process of iTRAQ-LC-MS/MS HC: healthy controls; HBV: hepatitis B virus infection; LC: liver cirrhosis; HCC: hepatocellular carcinoma.

Fractions were dried in a rotary vacuum concentrator and dissolved in buffer C (5% acetonitrile, 0.1% trifluoroacetic acid). Peptides were separated on a ZORBAX 300SB-C18 Column (0.1 3 15 mm, 5 mm, 300 Å; Microm, Auburn, CA) by applying a linear gradient of buffer D (95% acetonitrile, 4.99% water, 0.1% trifluoroacetic) from 5% to 35% over 70 min. Eluted peptides were analyzed using a QSTAR XL system (Applied Biosystems Inc., USA) connected to a 20AD HPLC system (Shimadzu, Kyoto, Japan). Eluted peptides were scanned over the range of 400–1,800 *m/z,* and then over the range of 100–2,000 *m/z*. The pathway of iTRAQ-LC-MS/MS analysis is shown in Figure 10.

### Data analysis

MS data were collected and identified using Analyst QS 1.1 software and ProteinPilot 3.0 software (Paragon Algorithm, Applied Biosystems Inc., USA). Raw data were analyzed using Applied Biosystems ProteinPilot software 4.5 (revision 1656). To qualify as differentially expressed proteins, they had to satisfy the following criteria: (1) proteins were present in the Swissprot database (SP_HUMAN.fas) with a reported confidence interval >95% (ProtScore >1.3); (2) proteins could be quantitated based on peak areas at *m/z* 113, 114, 115, 116, 117, 118, 119 and 121; (3) sampling error could be normalized using the bias correction function of the ProteinPilot software (Applied Biosystems Inc., USA); (4) Prot-Score (unused) >1.3, confidence interval >95%, EF <2, P <0.05, together with the neighboring time points protein value >1.2 or <0.8.

Binary logistic regression was used to assess the diagnostic value of CD14 alone and together with AFP for detecting HCC. CD14 and AFP levels were treated as independent variables. The value of the dependent variable was set to 0 for HCC or 1 for LC. The ENTER method was applied to screen variables, and the threshold for statistical significance was defined as p < 0.05.

### Bioinformatic analysis

All differentially expressed proteins identified in the discovery phase were analyzed using K-means clustering to characterize change trends; gene ontology (GO) classification to identify potentially disease-related biological processes, molecular functions and subcellular localization; and string network analysis (http://string-db.org) to analyze the interaction between identified proteins. These analyses were supplemented using data from the Human Protein Reference Database (HPRD; www.hprd.org).

### Western blot analysis

To validate a subset of differentially expressed proteins identified in iTRAQ-LC-MS/MS analysis as potential biomarkers for early HCC detection, the levels of these proteins in serum samples from different disease and control groups were analyzed by Western blotting. Protein samples (20 μg) were separated by 10% SDS-PAGE, and transferred to a PVDF membrane (0.45 mm thick). Membranes were stained with MemCode^TM^ Reversible Protein Stain Kit (Pierce, USA) to check for consistent protein loading and transfer. Membranes were then blocked with Tris-buffered saline containing Tween-20 (TBST, pH 8.0) at room temperature for 1 h, and incubated at 4 °C overnight with primary antibodies against candidate biomarkers CD14, gelsolin (GELS) and sulfhydryl oxidase 1 (QSOX1). Then membranes were incubated with horseradish peroxidase-conjugated secondary antibodies at room temperature for 1 h. Membranes were washed three times in TBST, incubated for 5 min with ECL plus chemiluminescent immunoblotting detection system (GE Healthcare) and then exposed to X-ray film. The intensity of each band was normalized to total band intensity for the given sample.

### Quantification of CD14 by ELISA

Levels of human CD14 in serum samples from 19 HCC patients and 18 LC patients were assayed using a commercial ELISA kit (Human sCD14 Quantikine ELISA, R&D, USA) according to the manufacturer’s instructions. Experiments were performed in duplicate.

## Abbreviations

HCC: hepatocellular carcinoma
HC: healthy controls
HBV: hepatitis B virus
LC: liver cirrhosis
AFP: alpha-fetoprotein
GELS: gelsolin
QSOX1: sulfhydryl oxidase 1
GO: gene ontology
iTRAQ: isobaric tagging for relative and absolute quantitation
ELISA: enzyme-linked immunosorbent assay
ROC: receiver operating characteristic
AUC: area under the curve
LC-MS/MS: liquid chromatography-tandem mass spectrometry
